# Supplementary material for: Multi‐dataset identification of innovative feature genes and molecular mechanisms in keratoconus
Source: J Cell Mol Med. 2024 Sep 19;28(18):e70079. doi: 10.1111/jcmm.70079 (PMC11412914; doi:10.1111/jcmm.70079)
Supplement: Supplementary file 2 — Material S2. [file JCMM-28-e70079-s002.zip › 7.GSEA analysis/6.SNORA74B/KEGG_PHENYLALANINE_METABOLISM.html]

Details for gene set KEGG\_PHENYLALANINE\_METABOLISM[GSEA]

|  || Dataset | SNORA74B.group.cls#h\_versus\_l |
| Phenotype | group.cls#h\_versus\_l |
| Upregulated in class | h |
| GeneSet | KEGG\_PHENYLALANINE\_METABOLISM |
| Enrichment Score (ES) | 0.36023366 |
| Normalized Enrichment Score (NES) | 1.0080895 |
| Nominal p-value | 0.47297296 |
| FDR q-value | 1.0 |
| FWER p-Value | 1.0 |
Table: GSEA Results Summary

  

Fig 1: Enrichment plot: KEGG\_PHENYLALANINE\_METABOLISM      
 Profile of the Running ES Score & Positions of GeneSet Members on the Rank Ordered List

  

| SYMBOL | TITLE | RANK IN GENE LIST | RANK METRIC SCORE | RUNNING ES | CORE ENRICHMENT || 1 | AOC2 | na | 747 | 0.534 | 0.1372 | Yes |
| 2 | TAT | na | 1441 | 0.422 | 0.2427 | Yes |
| 3 | MAOB | na | 1824 | 0.387 | 0.3469 | Yes |
| 4 | PAH | na | 4717 | 0.235 | 0.3335 | Yes |
| 5 | IL4I1 | na | 5832 | 0.198 | 0.3602 | Yes |
| 6 | GOT2 | na | 12459 | 0.054 | 0.1853 | No |
| 7 | ALDH3B1 | na | 12635 | 0.051 | 0.1956 | No |
| 8 | DDC | na | 15228 | 0.022 | 0.1275 | No |
| 9 | MAOA | na | 16871 | 0.006 | 0.0819 | No |
| 10 | MIF | na | 18896 | -0.016 | 0.0284 | No |
| 11 | GOT1 | na | 19922 | -0.028 | 0.0071 | No |
| 12 | PRDX6 | na | 21356 | -0.044 | -0.0211 | No |
| 13 | ALDH3A1 | na | 24095 | -0.082 | -0.0755 | No |
| 14 | ALDH3B2 | na | 27617 | -0.154 | -0.1310 | No |
| 15 | AOC3 | na | 32580 | -0.359 | -0.1671 | No |
| 16 | HPD | na | 32828 | -0.378 | -0.0617 | No |
| 17 | ALDH1A3 | na | 32981 | -0.391 | 0.0503 | No |
Table: GSEA details [plain text format]

  

Fig 2: KEGG\_PHENYLALANINE\_METABOLISM      
 Blue-Pink O' Gram in the Space of the Analyzed GeneSet

  

Fig 3: KEGG\_PHENYLALANINE\_METABOLISM: Random ES distribution      
 Gene set null distribution of ES for **KEGG\_PHENYLALANINE\_METABOLISM**
